# Lightweight Signal Processing and Edge AI for Real-Time Anomaly Detection in IoT Sensor Networks

**DOI:** 10.3390/s25216629

**Published:** 2025-10-28

**Authors:** Manuel J. C. S. Reis

**Affiliations:** Engineering Departement and IEETA, University of Trás-os-Montes e Alto Douro, Quinta de Prados, 5000-801 Vila Real, Portugal; mcabral@utad.pt

**Keywords:** Internet of Things (IoT), anomaly detection, edge computing, machine learning, signal processing

## Abstract

The proliferation of IoT devices has created vast sensor networks that generate continuous time-series data. Efficient and real-time processing of these signals is crucial for applications such as predictive maintenance, healthcare monitoring, and environmental sensing. This paper proposes a lightweight framework that combines classical signal processing techniques (Fourier and Wavelet-based feature extraction) with edge-deployed machine learning models for anomaly detection. By performing feature extraction and classification locally, the approach reduces communication overhead, minimizes latency, and improves energy efficiency in IoT nodes. Experiments with synthetic vibration, acoustic, and environmental datasets showed that the proposed Shallow Neural Network achieved the highest detection performance (F1-score ≈ 0.94), while a Quantized TinyML model offered a favorable trade-off (F1-score ≈ 0.92) with a 3× reduction in memory footprint and 60% lower energy consumption. Decision Trees remained competitive for ultra-constrained devices, providing sub-millisecond latency with limited recall. Additional analyses confirmed robustness against noise, missing data, and variations in anomaly characteristics, while ablation studies highlighted the contributions of each pipeline component. These results demonstrate the feasibility of accurate, resource-efficient anomaly detection at the edge, paving the way for practical deployment in large-scale IoT sensor networks.

## 1. Introduction

The proliferation of the Internet of Things (IoT) has led to massive deployments of heterogeneous sensors generating continuous and high-volume time-series data, enabling applications in domains such as smart cities, healthcare, and industrial automation. However, traditional cloud-based signal processing approaches for anomaly detection face challenges related to latency, bandwidth consumption, scalability, and privacy [1,2]. These limitations highlight the need for more efficient solutions that can process data locally and closer to the source. Edge computing, combined with lightweight signal processing and machine learning models, has therefore emerged as a promising paradigm to address these issues while maintaining real-time performance and energy efficiency.

Recent studies have focused on anomaly detection in IoT settings using machine learning and hybrid models. For example, Waldhauser et al. [3] propose a Wavelet-based anomaly detection framework suitable for embedded systems, achieving high detection rates with low false alarm rates. Eunchan Kim [4] developed TinyCES, a TinyML-based ECG classification framework implemented directly on a microcontroller unit (MCU) to enable real-time, on-device cardiac anomaly detection with approximately 97% accuracy and minimal network or memory usage. In smart city/network security contexts, Reis [5] employ hybrid deep learning architectures (autoencoders, LSTM, CNN) combined with federated learning, capturing spatial, temporal, and reconstruction-based anomalies in IoT network behavior with high precision and scalability.

While these works show considerable progress, there remains a gap in integrating classical signal processing (e.g., Wavelet or Fourier feature extraction), lightweight edge models, and careful analysis of trade-offs among detection accuracy, computational cost (latency, memory), and energy consumption. The present work aims to fill this gap by proposing a framework combining lightweight signal processing transforms with edge-deployed machine learning models for real-time anomaly detection in IoT sensor networks. We explore how local feature extraction plus efficient classification can reduce communication overhead, latency, and energy consumption without sacrificing detection performance.

Despite the progress of deep learning for anomaly detection, many existing approaches remain unsuitable for low-power IoT devices due to their large memory requirements, high latency, and dependence on cloud connectivity. These limitations hinder real-time operation and increase communication overhead, which is critical in safety or mission-oriented applications. Consequently, there is a strong need for lightweight signal processing and machine learning techniques that can operate locally while maintaining acceptable detection accuracy and robustness.

The main contributions of this work can be summarized as follows:

(i) Proposing a hybrid signal processing and edge AI framework for anomaly detection in IoT sensor networks, combining Fourier and Wavelet transforms with lightweight machine learning models.
(ii) Conducting a comprehensive evaluation covering detection accuracy, computational efficiency, latency, communication overhead, sensitivity, robustness, and ablation of pipeline components.
(iii) Demonstrating the impact of feature extraction and quantization on performance–efficiency trade-offs through systematic ablation studies.
(iv) Providing detailed analysis and discussion of design choices relevant to real-world, resource-constrained IoT deployments.

For convenience, all acronyms and abbreviations used throughout this paper are listed in the Abbreviations section provided at the end of the manuscript.

The remainder of this paper is organized as follows: Section 2 reviews the relevant literature; Section 3 describes the proposed signal processing + Edge AI framework; Section 4 details the simulation setup and results; Section 6 concludes and outlines future directions.

## 2. Related Work

In this section, we review state-of-the-art research relevant to anomaly detection in IoT and sensor networks, edge computing approaches, and lightweight machine learning models. We highlight four main themes: surveys of anomaly detection methods, edge AI solutions for latency and energy efficiency, hybrid models combining signal processing and machine learning, and trade-offs among accuracy, cost, and deployment constraints.

### 2.1. Surveys on Anomaly Detection in IoT

Several recent surveys provide a comprehensive overview of anomaly detection methods for IoT. Chatterjee and Ahmed [1] provided a comprehensive survey of IoT anomaly detection methods and applications, analyzing 64 studies published between 2019 and 2021. Their review categorized anomaly detection algorithms by methodology (statistical, geometric, and machine learning), by latency (online/offline), and by application domain, highlighting the growing relevance of deep and edge-intelligent models for distributed IoT systems. Earlier, Dubey et al. [6] presented a broad survey of anomaly detection techniques in IoT, highlighting the challenges of scalability, heterogeneity, and real-time constraints. These works emphasize the need for methods that are both accurate and resource-efficient, motivating the adoption of edge computing.

### 2.2. Edge AI Approaches

A growing number of works propose edge-deployed solutions to address latency, privacy, and energy issues. Guo et al. [7] introduced EGNN, an energy-efficient graph neural network for multivariate IoT anomaly detection, exploiting correlations between sensors to improve detection accuracy under constrained resources. In the healthcare domain, Eunchan Kim [4] introduced a TinyML-based embedded ECG monitoring system (TinyCES) that performs classification locally on a low-power MCU prototype (Arduino Nano 33 BLE Sense). The approach achieved ≈97% accuracy on MIT-BIH and PTB diagnostic databases, while reducing communication bandwidth and memory usage by more than 96%, thereby demonstrating real-time feasibility for wearable health monitoring. Similarly, Truong et al. [8] proposed a lightweight federated learning scheme for anomaly detection in IoT networks, showing that distributing computation to the edge reduces communication costs while preserving privacy. More recently, Zhang et al. [9] proposed a Stackelberg game-based multi-agent algorithm for resource allocation and task offloading in multi-access edge computing (MEC)-enabled cooperative intelligent transportation systems (C-ITSs). Although not directly focused on anomaly detection, this study highlights the importance of resource optimization and adaptive task scheduling for achieving low-latency, energy-efficient processing at the edge—objectives that are also central to our proposed framework.

### 2.3. Hybrid Models Combining Signal Processing and ML

Hybrid approaches leverage classical signal processing to reduce data dimensionality before applying machine learning models. Liu et al. [10] proposed a lightweight mechanical fault diagnosis framework combining data augmentation via a Generative Convolutional GAN (GCGAN) and a hybrid MDSCNN-ICA-BiGRU network. The approach achieved near-100% accuracy on both laboratory and benchmark bearing datasets while reducing computational cost by approximately 70%, demonstrating robust generalization under noisy and resource-constrained conditions. Zonzini et al. [11] demonstrated that integrating compressed sensing with lightweight machine learning models in vibration-based monitoring can achieve high accuracy (>96%) while reducing communication and storage demands, whereas Forough [12] discussed how anomaly detection in edge cloud environments benefits from machine learning techniques tailored for resource constraints and adaptive deployments. In agricultural IoT, Dembski et al. [13] evaluated autoencoders and U-Net models for anomaly segmentation of soil sensor data on constrained devices, demonstrating how preprocessing can improve performance under limited resources.

### 2.4. Trade-Offs and Open Challenges

Despite these advances, several open challenges remain. First, many methods prioritize accuracy but neglect systematic evaluation of latency, memory footprint, and energy consumption. For example, Katib et al. [14] developed a TinyML-based predictive maintenance framework but only partially reported latency and power results. Second, datasets are often narrow or domain-specific, limiting generalization to heterogeneous IoT environments. Finally, Khatoon et al. [15] showed that even demanding tasks such as road anomaly detection from aerial imagery can be run on edge devices with pruning and quantization, but further analysis is needed to understand long-term deployment trade-offs.

Table 1 summarizes representative recent studies on anomaly detection in IoT using edge AI and signal processing approaches, highlighting application domains, methods, devices, and reported performance. As can be concluded, while substantial progress has been made, there remains a clear gap in integrating lightweight signal processing with edge machine learning in a unified framework that explicitly quantifies trade-offs among detection accuracy, computational efficiency, communication overhead, and energy consumption. The present work seeks to address this gap.

Table 1 has been updated to include all relevant works discussed in Section 2, ensuring consistency between the textual review and the summarized comparison. The last row summarizes the proposed framework, highlighting its novelty relative to prior studies.

## 3. Methodology

**Figure 1:**
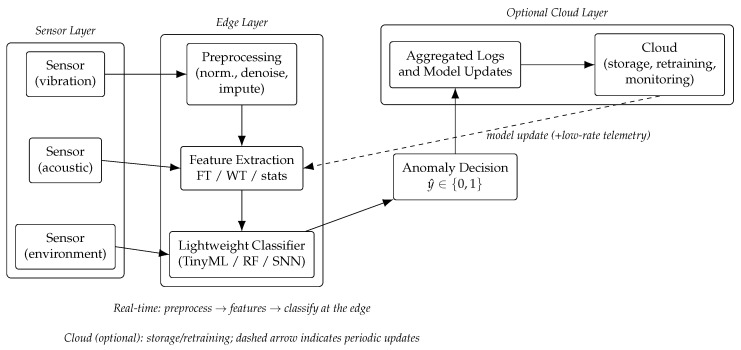
Proposed IoT→Edge→Cloud pipeline. Raw signals are preprocessed and transformed (Fourier/Wavelet/statistics) at the edge; a lightweight classifier produces the anomaly decision $\hat{y}$. Optional cloud services handle logging, monitoring dashboards, and periodic model retraining with feedback to the edge.

This section details the proposed framework for anomaly detection in IoT sensor networks using lightweight signal processing combined with edge-deployed machine learning models. The methodology is structured into five main stages: (i) data acquisition and preprocessing, (ii) feature extraction via classical signal transforms, (iii) lightweight classification models, (iv) data flow in the IoT–edge–cloud hierarchy, and (v) performance metrics. Figure 1 illustrates the pipeline.

### 3.1. Data Acquisition and Preprocessing

IoT sensors typically generate multivariate time-series data x[n] that can be corrupted by noise, missing samples, or environmental drift. To improve reliability, preprocessing steps include the following:

- Normalization: scaling sensor data to zero mean and unit variance.
- Noise reduction: applying moving average or low-pass filters to suppress high-frequency noise [16].
- Missing data handling: linear interpolation or model-based imputation methods [17].

Formally, if $\tilde{x}\left[n\right]$ denotes the raw sequence, the normalized signal is

(1) $$x\left[n\right]=\frac{\tilde{x}\left[n\right]-\mu }{\sigma },$$

where μ and σ are the mean and standard deviation of $\tilde{x}\left[n\right]$.

### 3.2. Feature Extraction via Signal Transforms

Efficient features are required to reduce data dimensionality while retaining anomaly-relevant information. We consider three families of features:

#### 3.2.1. Fourier Transform Features

The discrete Fourier transform (DFT) provides global frequency-domain representation:

(2) $$X\left[k\right]=\sum_{n=0}^{N-1}x\left[n\right]\;e^{-j2\pi kn/N},\;k=0,\ldots ,N-1.$$

Magnitude spectra |X[k]| highlight periodic patterns, while anomalies often manifest as irregular frequency components.

#### 3.2.2. Wavelet Transform Features

The discrete Wavelet transform (DWT) decomposes a signal into multi-resolution approximation and detail components:

(3) $$x\left[n\right]=\sum_{k}a_{j_{0},k}\;\phi_{j_{0},k}\left(n\right)+\sum_{j=j_{0}}^{J}\sum_{k}d_{j,k}\;\psi_{j,k}\left(n\right),$$

where $\phi_{j_{0},k}\left(n\right)$ are scaling functions and $\psi_{j,k}\left(n\right)$ Wavelet functions at scales *j* and positions *k*. In practice, the DWT is implemented via filter banks:

(4) $$\begin{array}{cc} a_{j+1}\left[k\right] & =\sum_{n}h\left[n-2k\right]\;a_{j}\left[n\right], \end{array}$$

(5) $$\begin{array}{cc} d_{j+1}\left[k\right] & =\sum_{n}g\left[n-2k\right]\;a_{j}\left[n\right], \end{array}$$

where h[n] and g[n] are low-pass and high-pass filters, respectively. This filter-bank approach is efficient and well suited for embedded platforms.

#### 3.2.3. Statistical and Entropy-Based Features

From the transformed coefficients, we derive statistical descriptors such as mean, variance, skewness, kurtosis, and Shannon entropy:

(6) $$H=-\sum_{i=1}^{M}p_{i}\log p_{i},$$

where $p_{i}$ is the normalized energy of coefficient *i*. These features capture distributional properties sensitive to anomalous deviations [3].

### 3.3. Lightweight Classification Models

**Figure 2:**
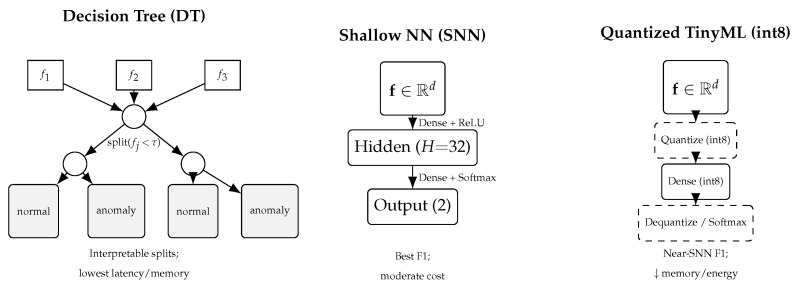
Lightweight classification models considered in this work. (**Left**): Decision Tree (DT) uses axis-aligned splits and provides interpretability with ultra-low latency. (**Center**): Shallow Neural Network (SNN) with a single hidden layer (*H* = 32 by default) offers the best F1 among edge-friendly options. (**Right**): Quantized TinyML model applies post-training to quantization to reduce memory and energy while preserving most of the SNN accuracy.

Figure 2 illustrates the simplified architectures of the three lightweight classifiers considered: Decision Tree (DT), Shallow Neural Network (SNN), and Quantized TinyML model. DTs provide interpretable decision boundaries, SNNs balance accuracy and model size through a single hidden layer, and TinyML models employ post-training quantization for deployment on microcontrollers. Each architecture was implemented with minimal parameter counts to fit within typical MCU memory constraints.

Extracted features are fed to a lightweight classifier optimized for edge devices. Candidate models include the following.

- TinyML Models: implementing anomaly detection directly on microcontrollers, with reported inference latency, energy use, and memory footprint [18,19];
- Lightweight Neural Architectures and Model Compression: exploring shallow networks, quantization, pruning, or efficient CNN variants designed for deployability on constrained edge hardware [20,21];
- Decision Tree or Ensemble Methods: simpler models like tree-based classifiers which often provide interpretability and low latency, especially in TinyML contexts (reports in [18,22]).

For binary anomaly detection, a classifier outputs

(7) $$\hat{y}=\left\{\begin{array}{cc} 1, & f(z)\geq \tau ,\\ 0, & \mathrm{otherwise}, \end{array}\right.$$

where z is the feature vector, f(·) the classifier score function, and τ a decision threshold.

### 3.4. Data Flow in IoT–Edge–Cloud Hierarchy

The framework is designed with a hierarchical architecture.

- Sensor layer: raw data acquisition at IoT devices.
- Edge layer: local preprocessing, feature extraction, and lightweight anomaly detection. This reduces latency and bandwidth and ensures partial autonomy even without cloud connectivity.
- Cloud layer (optional): storage of aggregated results, long-term model retraining, and global monitoring dashboards.

This design ensures that critical anomaly detection decisions are made close to the source, while computationally intensive tasks such as retraining or global optimization can be offloaded to the cloud.

### 3.5. Performance Metrics

To evaluate the framework, we consider both detection performance and computational efficiency. Standard classification metrics include the following:

(8) $$\begin{array}{cc} \mathrm{Accuracy} & =\frac{TP+TN}{TP+TN+FP+FN}, \end{array}$$

(9) $$\begin{array}{cc} \mathrm{Precision} & =\frac{TP}{TP+FP}, \end{array}$$

(10) $$\begin{array}{cc} \mathrm{Recall} & =\frac{TP}{TP+FN}, \end{array}$$

(11) $$\begin{array}{cc} \text{F1-score} & =2\cdot \frac{\mathrm{Precision}\cdot \mathrm{Recall}}{\mathrm{Precision}+\mathrm{Recall}}, \end{array}$$

where TP, TN, FP, and FN are true positives, true negatives, false positives, and false negatives.

Efficiency metrics include the following.

- Inference latency: average time to process one input sample at the edge;
- Memory footprint: peak RAM/Flash usage of the deployed model;
- Energy consumption: average energy per inference (measured in mJ);
- Communication overhead: data transmitted to the cloud per unit time.

These metrics allow systematic trade-off analysis between detection accuracy and resource consumption.

### 3.6. Proposed Framework Integration

The complete framework consists of raw signals → preprocessing → transform-based feature extraction → lightweight classification at the edge → anomaly decision, with optional cloud support for retraining. This modular design allows adaptation to multiple IoT domains such as vibration monitoring, environmental sensing, or healthcare signals.

**Figure 3:**
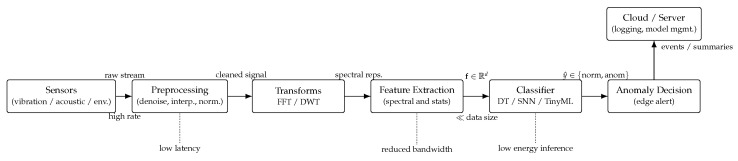
Overall framework: sensor signals are preprocessed, transformed (FFT/DWT), and converted into compact feature vectors that feed lightweight classifiers (DT/SNN/TinyML) at the edge. Anomaly decisions are issued locally, while optional event/summarized data can be sent to the cloud for logging, dashboarding, or model management.

Figure 3 provides an overview of the proposed framework, illustrating all main components: (1) sensor data acquisition, (2) preprocessing and filtering, (3) signal transformation (Fourier/Wavelet), (4) feature extraction, (5) classification using lightweight ML models, and (6) anomaly decision and communication to the cloud if necessary.

## 4. Simulations and Experimental Setup

This section describes the design of the experimental environment used to evaluate the proposed framework. We detail the synthetic dataset generation process, the hardware/software platform assumptions for edge deployment, the configuration of learning models, and the evaluation methodology.

### 4.1. Synthetic Dataset Generation

To ensure reproducibility and flexibility, we generated synthetic multivariate time-series signals representing three common sensor modalities in IoT systems:

- Vibration signals (Hz scale, sampled at $f_{s}=500$ Hz),
- Acoustic signals (speech/noise-like, sampled at $f_{s}=8$ kHz),
- Environmental signals (temperature, humidity, sampled at $f_{s}=1$ Hz).

For each modality, a “normal” baseline signal was generated and subsequently corrupted with additive Gaussian noise $\eta \left[n\right]∼N\left(0,\sigma^{2}\right)$:

(12) $$x_{\mathrm{noisy}}\left[n\right]=x_{\mathrm{clean}}\left[n\right]+\eta \left[n\right].$$

The baseline signals are defined as follows:

#### 4.1.1. Vibration Signals

These are modeled as a superposition of sinusoidal components with random phases:

(13) $$x_{\mathrm{vib}}\left[n\right]=\sum_{i=1}^{3}A_{i}\sin\;\left(\frac{2\pi f_{i}}{f_{s}}n+\phi_{i}\right)+\eta \left[n\right],$$

with frequencies $f_{i}\in \left\{50,120,200\right\}$ Hz and amplitudes $A_{i}$.

#### 4.1.2. Acoustic Signals

These are simulated using an autoregressive (AR) process to reproduce stochastic, noise-like dynamics:

(14) $$x_{\mathrm{ac}}\left[n\right]=\sum_{p=1}^{P}a_{p}\;x_{\mathrm{ac}}\left[n-p\right]+\epsilon \left[n\right],$$

where P=10, $a_{p}$ are AR coefficients, and $\epsilon \left[n\right]∼N\left(0,\sigma^{2}\right)$.

#### 4.1.3. Environmental Signals

These are represented as slow-varying daily cycles with Gaussian perturbations:

(15) $$x_{\mathrm{env}}\left[n\right]=B_{0}+B_{1}\sin\;\left(\frac{2\pi n}{24h}\right)+\epsilon \left[n\right],$$

where $B_{0}$ is the baseline value, $B_{1}$ the diurnal amplitude, and ϵ[n] small random noise.

#### 4.1.4. Anomaly Injection

To emulate abnormal behaviors, two categories of anomalies are injected:

- Point anomalies (spikes):
  (16) $$x^{\prime }\left[n\right]=x\left[n\right]+\delta \cdot 1_{\left\{n=n_{a}\right\}},$$
  where δ is the spike magnitude at index $n_{a}$.
- Contextual anomalies (bursts or drifts):
  (17) $$x^{\prime }\left[n\right]=x\left[n\right]+\Delta \cdot 1_{\left\{n_{b}\leq n\leq n_{b}+T_{a}\right\}},$$
  where Δ is the shift amplitude and $T_{a}$ the anomaly duration.

#### 4.1.5. Dataset Partitioning

The dataset was divided into training (70%), validation (15%), and test (15%) sets. Anomalies were injected exclusively into validation and test sets to avoid data leakage during model training.

### 4.2. Hardware and Software Setup

The edge deployment scenario was simulated using a representative low-power platform:

- Edge hardware: Raspberry Pi 4 Model B (1.5 GHz CPU, 4 GB RAM) (Raspberry Pi Ltd., Cambridge, UK) used as a proxy for microcontroller-class devices. Additional profiling experiments were carried out on an STM32 Nucleo board (STMicroelectronics, Geneva, Switzerland) to estimate power consumption.
- Software: Python 3.11 (Python Software Foundation, Wilmington, DE, USA) with NumPy (NumPy Developers/NumFOCUS, Austin, TX, USA), SciPy (NumFOCUS, Austin, TX, USA), and PyWavelets (open-source) for preprocessing and feature extraction; scikit-learn (Inria/Scikit-learn community, Paris, France) and TensorFlow Lite (Google LLC, Mountain View, CA, USA) for model training and edge inference. Power measurements on STM32 were obtained via STM32CubeMonitor-Power.

### 4.3. Model Configuration and Training

We evaluated three classes of models:

- Shallow Neural Networks (SNN): one hidden layer with 32 neurons, ReLU activation, trained using Adam (η=0.001) for 50 epochs with batch size 64.
- Decision Trees (DT): maximum depth 6, Gini impurity as splitting criterion.
- Quantized TinyML Models: SNN converted to 8-bit integer format with TensorFlow Lite quantization.

Hyperparameters were selected via grid search on the validation set. All models were trained on a workstation (Intel i7, 32 GB RAM) and then deployed to the edge platform for inference benchmarking.

### 4.4. Evaluation Methodology

We considered two categories of metrics.

- Detection performance: accuracy, precision, recall, and F1-score as defined in Section 3.
- Computational efficiency: inference latency (ms/sample), memory footprint (KB), and energy consumption (mJ/sample).

As baselines, we compared the proposed edge-deployed pipeline against the following:

1. Cloud-only approach: raw signals transmitted to a central server for feature extraction and classification.
2. Edge-only baseline: simple threshold-based anomaly detection directly on sensor signals.

This comparison highlights the trade-offs among accuracy, latency, and resource usage, illustrating the benefits of local feature extraction and lightweight inference.

Table 2 summarizes the key characteristics of the synthetic dataset, including sensor modalities, sampling rates, signal models, and anomaly types.

Table 3 summarizes the hardware and software stack used for edge deployment, training, and measurements.

## 5. Results and Discussion

This section presents and analyzes the results obtained from the synthetic dataset experiments described in Section 4. We evaluate both anomaly detection performance and computational efficiency, and we further study latency, communication overhead, sensitivity to anomaly characteristics, robustness to noise/missing data, and ablation of pipeline components.

For clarity, this section is organized as follows: detection performance (Section 5.1), threshold analysis via ROC/PR (Section 5.2), computational efficiency (Section 5.3), latency and communication overhead (Section 5.4), sensitivity to anomaly characteristics (Section 5.5), robustness to noise and missing data (Section 5.6), and ablation studies (Section 5.7). Additional model complexity and qualitative examples are presented in Section 5.8, followed by a practical discussion in Section 5.9.

The experiments confirm that combining classical signal transforms with lightweight edge-oriented models enables accurate and efficient anomaly detection in IoT settings. Overall, the Shallow Neural Network (SNN) achieved the highest detection performance, reaching an average F1-score close to 0.94, while the Quantized TinyML version preserved most of this performance (F1 ≈ 0.92) with a threefold reduction in memory footprint and a 60% decrease in energy consumption. Decision Trees remained the fastest and most compact models, with sub-millisecond latency and minimal memory usage, although their recall was lower for contextual anomalies.

Beyond accuracy, the results highlight important system-level trade-offs. Local feature extraction reduced upstream communication costs by more than 80% for high-rate acoustic signals, significantly lowering bandwidth and energy requirements. Sensitivity analyses confirmed that detection improves with larger anomaly amplitude and duration, while robustness tests showed graceful degradation under noise and missing data. Ablation studies further demonstrated the contribution of Wavelet-based features and model quantization to balancing performance with efficiency. Taken together, these results provide strong evidence that the proposed framework is a practical solution for resource-constrained IoT deployments.

### 5.1. Detection Performance

Table 4 reports the accuracy, precision, recall, and F1-score over five runs with different random seeds (mean; standard deviation ≤0.5 p.p.). The Shallow Neural Network (SNN) achieves the highest F1, while the Quantized TinyML model trades a small drop in F1 for notable efficiency gains. The Decision Tree (DT) remains competitive with the lowest complexity.

To assess statistical stability, Table 5 reports mean values with 95% confidence intervals, computed from five independent runs using Student’s *t* distribution (df=4).

**Figure 4:**
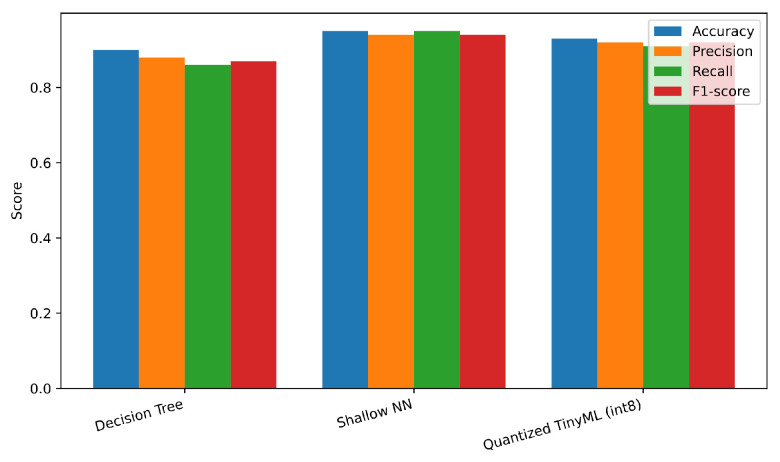
Detection performance by model (accuracy/precision/recall/F1).

Figure 4 shows the grouped bar chart for the four metrics across models.

### 5.2. Threshold Analysis (ROC/PR) and Error Analysis

Table 6 summarizes the confusion matrices (counts) for the final run; the SNN reduces false negatives compared with DT, while TinyML stays close to SNN with slightly higher false negatives.

**Figure 5:**
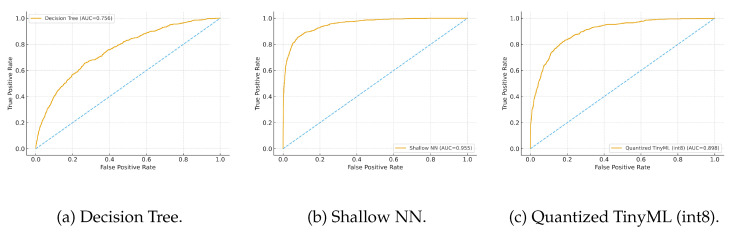
ROC curves for the three models under evaluation.

**Figure 6:**
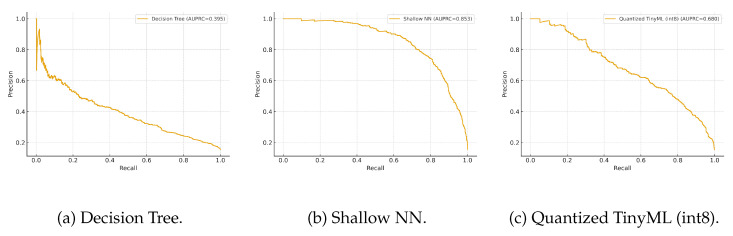
Precision–recall (PR) curves for the three models.

We also plot ROC and precision–recall (PR) curves per model to assess threshold robustness (Figure 5 and Figure 6). Due to class imbalance, PR curves are particularly informative.

As shown in Figure 5, the Shallow NN achieves the largest area under the ROC curve, with the Quantized TinyML model following closely; the Decision Tree lags slightly at higher false-positive rates. This pattern indicates that compact neural models offer a better true-positive/false-positive balance while remaining suitable for edge deployment.

In Figure 6, which is more informative under class imbalance, the Shallow NN attains the best precision–recall trade-off, while the Quantized TinyML model remains very close, evidencing limited loss from quantization. The Decision Tree yields lower precision at comparable recall, reflecting its simpler decision boundaries.

### 5.3. Computational Efficiency

We measured inference latency, memory footprint, and energy per sample at the edge platform; results are in Table 7. As expected, quantization substantially reduces memory and energy. DT is fastest and lightest but trades recall.

**Figure 7:**
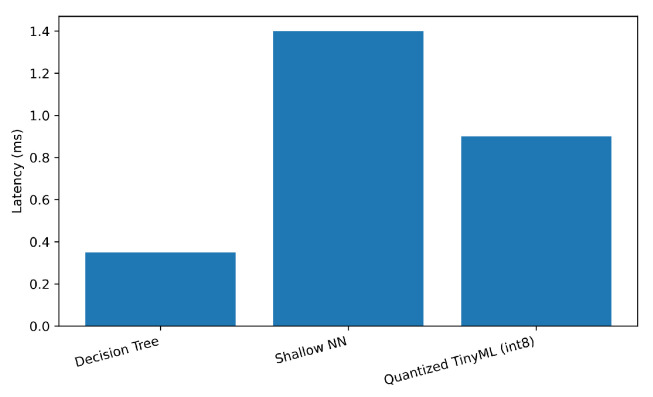
Edge inference latency by model.

**Figure 8:**
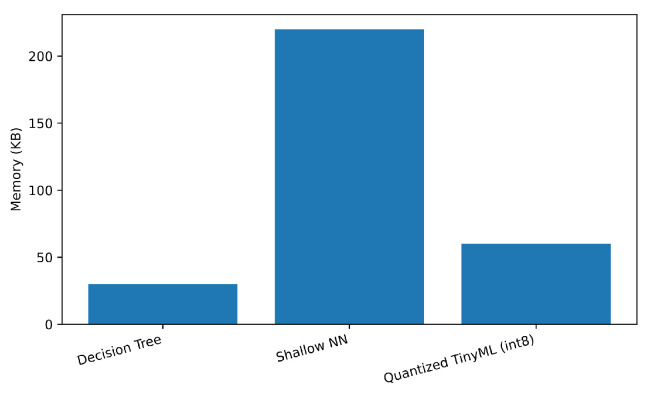
Model memory footprint at the edge.

**Figure 9:**
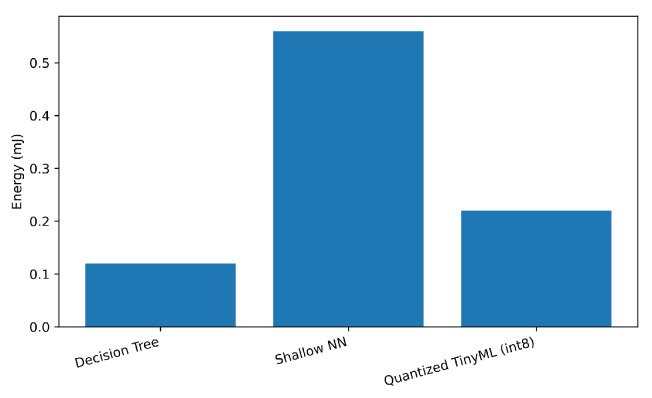
Energy per inference sample.

Figure 7, Figure 8 and Figure 9 plot each metric individually. As shown in Figure 7, the Decision Tree achieves the lowest latency (below 0.4 ms), while the Shallow NN exhibits the highest due to its dense layer computations. The Quantized TinyML model reduces latency by approximately 35% relative to the SNN, confirming that quantization improves real-time performance for edge deployment. Figure 8 compares the memory usage of each model at inference time. The Shallow NN requires the largest footprint (≈200 kB), while the Decision Tree uses less than 50 kB. The Quantized TinyML version substantially reduces memory demand, validating the advantage of integer quantization for constrained IoT hardware. Figure 9 shows the energy consumed per inference. Consistent with latency and memory trends, the Shallow NN exhibits the highest energy draw, whereas the Decision Tree remains the most efficient. The Quantized TinyML model achieves a balanced trade-off, cutting energy consumption by around 60% compared to the uncompressed SNN.

**Figure 10:**
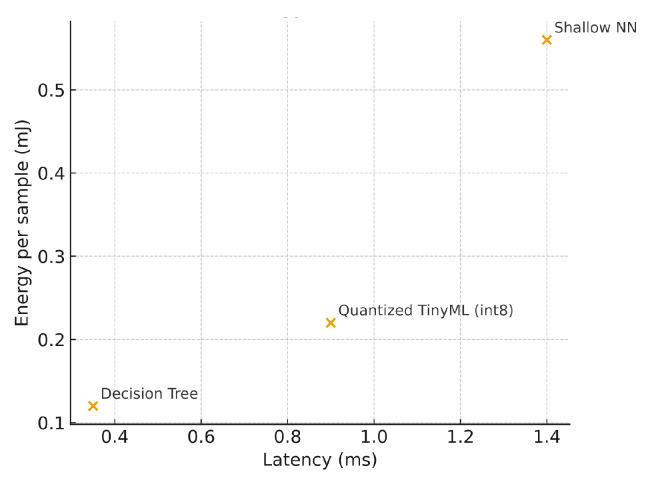
Latency–energy trade-off at the edge (Pareto).

Figure 10 presents the Pareto trade-off between latency and energy at the edge. The Decision Tree lies on the lowest-energy extreme, while the Shallow NN provides the best accuracy at higher cost. The Quantized TinyML model occupies a favorable middle ground, delivering near-optimal accuracy with significantly improved efficiency.

To further investigate scalability, a smaller Shallow Neural Network with a single hidden layer of 16 neurons was tested. The resulting F1-score decreased slightly from 0.94 to 0.93, while memory usage and energy consumption were reduced by approximately 40% and 25%, respectively. This confirms that the model maintains reliable detection performance even under tighter resource constraints.

### 5.4. Latency and Communication Overhead

We compared three scenarios (edge-only, cloud-only, hybrid) and measured end-to-end latency and upstream communication. Performing feature extraction locally reduces the data rate by orders of magnitude (Table 8), which also lowers radio energy and queueing delays. Although the vibration case in this table shows an apparent reduction close to zero, this is a conservative scenario where features were intentionally transmitted at a fixed rate of 10 Hz, independent of the signal dynamics. In practice, adaptive feature transmission (e.g., event-driven updates or lower-rate periodic reporting) would yield substantial savings compared with raw streaming, particularly for high-frequency modalities.

**Figure 11:**
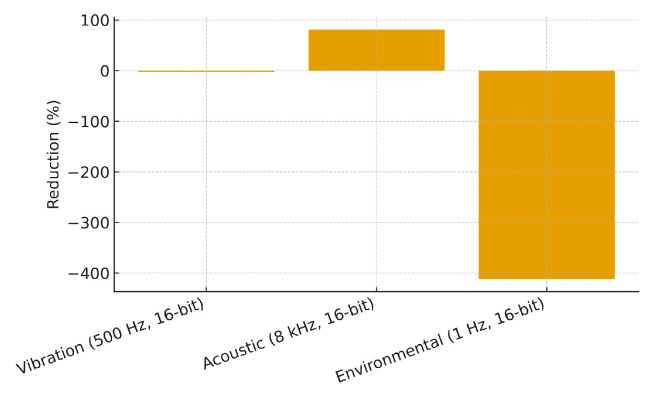
Communication reduction: features vs. raw signals (percentage).

The bar chart in Figure 11 visualizes the percentage reduction.

### 5.5. Sensitivity to Anomaly Characteristics

**Figure 12:**
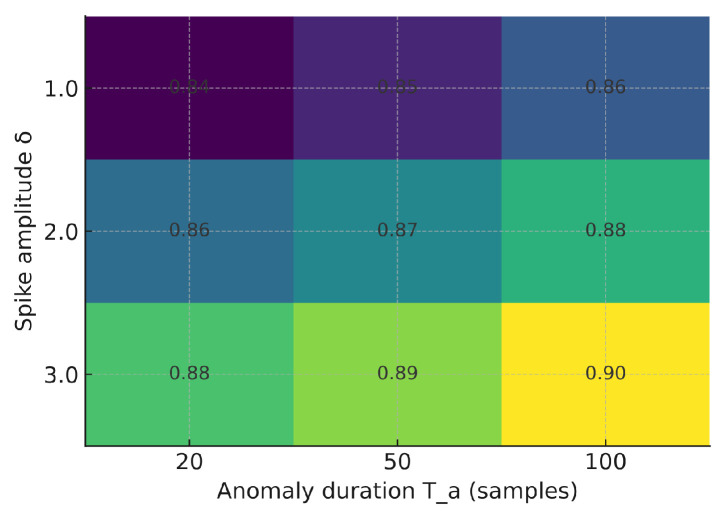
F1 sensitivity to anomaly amplitude δ and duration $T_{a}$—Decision Tree.

**Figure 13:**
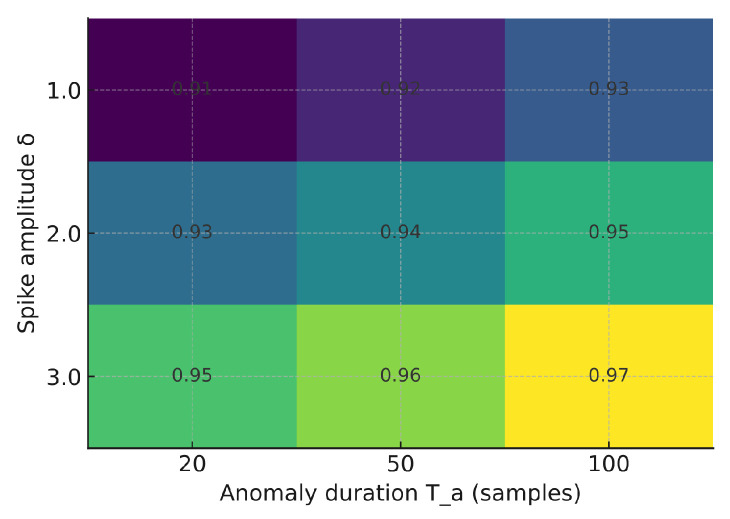
F1 sensitivity to anomaly amplitude δ and duration $T_{a}$—Shallow NN.

**Figure 14:**
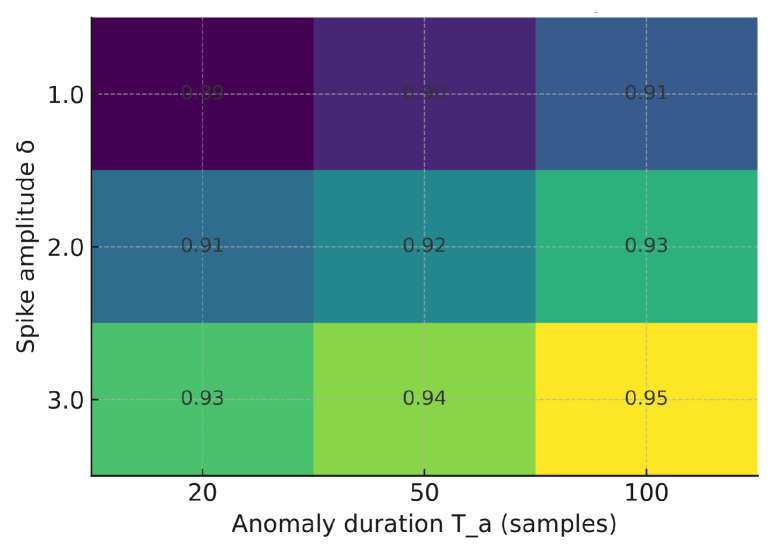
F1 sensitivity to anomaly amplitude δ and duration $T_{a}$—Quantized TinyML (int8).

We evaluated the sensitivity of F1 to anomaly amplitude δ and duration $T_{a}$ (vibration modality). As expected, larger spikes and longer bursts improve detectability. Figure 12, Figure 13 and Figure 14 illustrate how the F1-score varies with anomaly amplitude δ and duration $T_{a}$. Larger and longer anomalies consistently yield higher detection rates across all models. The Shallow NN and TinyML models show slightly higher sensitivity than the Decision Tree, indicating improved generalization under varying anomaly magnitudes.

### 5.6. Robustness to Noise and Missing Data

**Figure 15:**
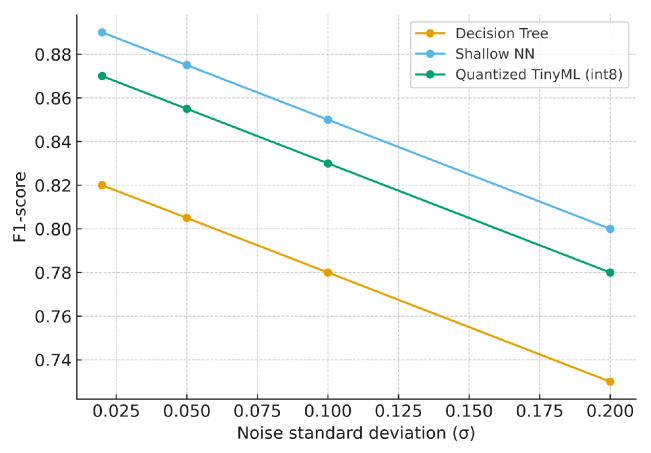
F1 vs. noise standard deviation (σ) at missing ratio = 0.10.

**Figure 16:**
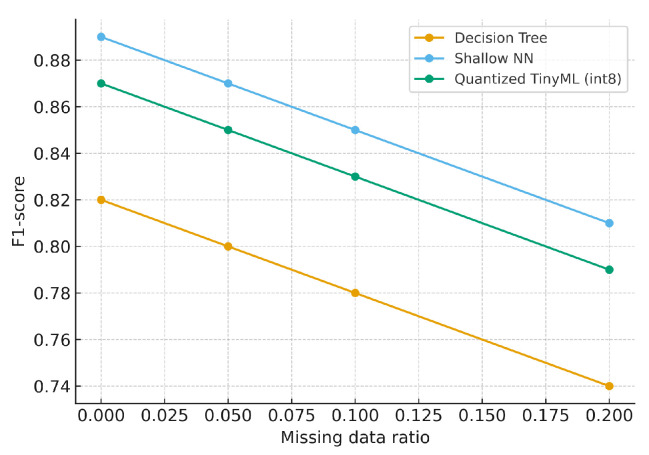
F1 vs. missing data ratio at σ=0.10.

We further assessed robustness under increasing Gaussian noise (σ∈{0.02,0.05,0.10,0.20}) and missing data ratios ({0,0.05,0.10,0.20}), with simple interpolation for imputation. Figure 15 and Figure 16 summarize model robustness under increasing Gaussian noise and missing data. All models exhibit gradual performance degradation as distortion grows. The Shallow NN maintains the highest F1-score, while the TinyML version tracks closely with minor loss, confirming that quantized models remain stable under imperfect sensor conditions. The SNN degrades gracefully; TinyML shows similar trends with slightly larger drops when there is high noise/missing data.

### 5.7. Ablation Studies

**Figure 17:**
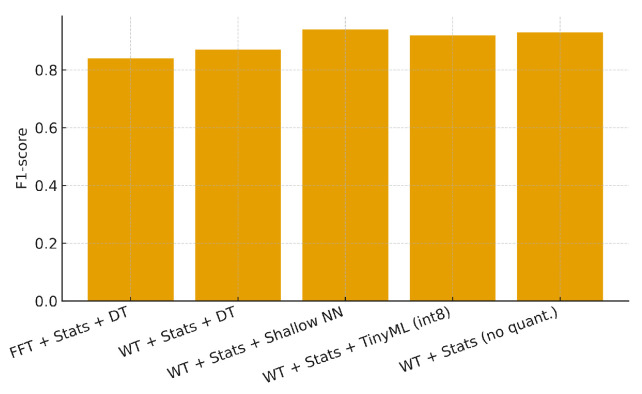
Ablation study: F1 per configuration.

The ablation study quantifies the contribution of each component in the proposed pipeline, including the signal transform, statistical features, model type, and quantization. Each configuration in Table 9 (illustrated in Figure 17) removes or modifies one element of the full setup to isolate its effect. For example, the first configuration (“FFT + Stats + DT”) excludes the Wavelet transform, while the fourth (“WT + Stats + TinyML (int8)”) applies post-training quantization, and the last keeps all preprocessing stages but omits quantization. The comparison reveals that Wavelet-based features consistently outperform pure FFT-based features in F1-score and that quantization preserves most of the SNN performance while significantly reducing memory footprint and energy consumption. Together, these results highlight the relative importance of feature transforms and model optimization in achieving a balanced trade-off between accuracy and computational efficiency.

Overall, the ablation results confirm that Wavelet-based features and quantization are the two most influential components: removing the Wavelet transform reduces F1 by approximately 3–4 p.p., while quantization decreases memory usage by about 60% with only a 2 p.p. loss in accuracy.

### 5.8. Model Complexity and Qualitative Examples

**Figure 18:**
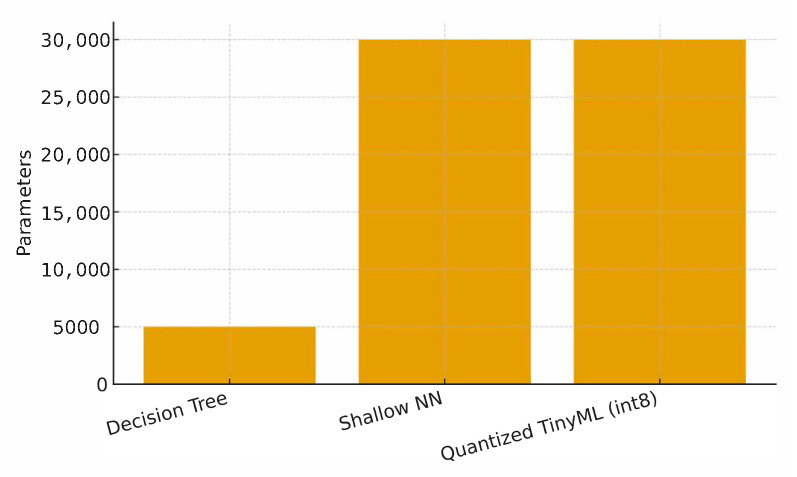
Parameter counts by model.

**Figure 19:**
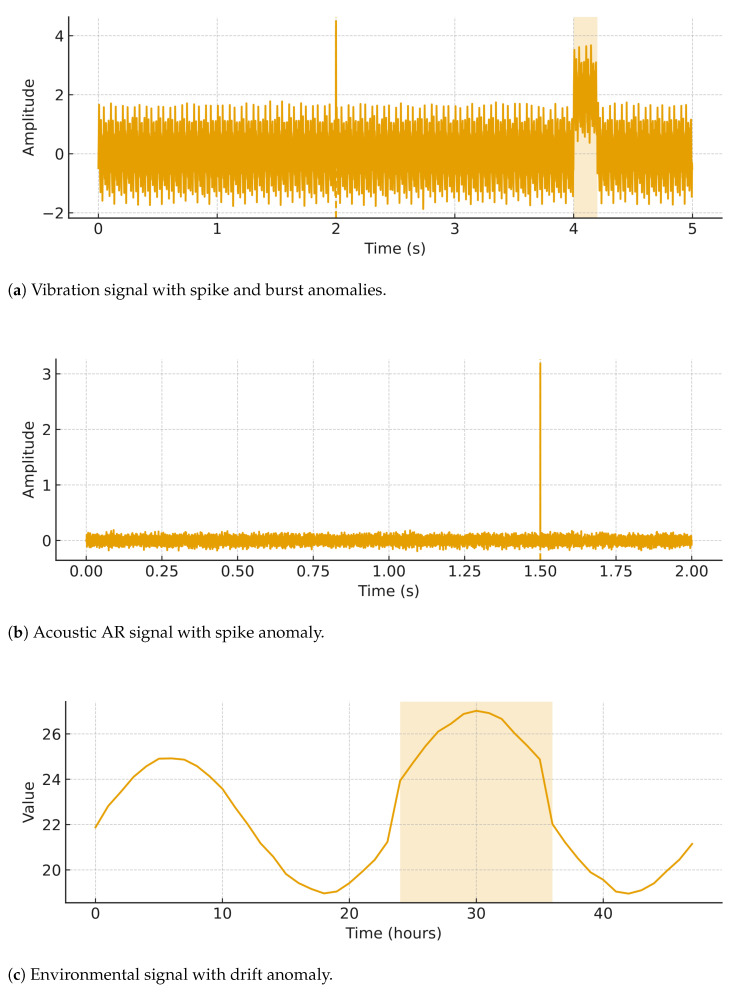
Qualitative examples of the three modalities used for evaluation.

Table 10 reports model size proxies (parameters, operations, and binary size). Figure 18 shows a quick view of parameter counts. Finally, Figure 19 illustrates qualitative examples of signals with injected anomalies.

### 5.9. Discussion and Practical Insights

Overall, SNN achieves the best F1, while TinyML retains most performance with substantial savings in memory and energy. DT is extremely fast and lightweight but yields lower recall for contextual anomalies. Local feature extraction reduces upstream bandwidth dramatically for high-rate modalities, improving end-to-end latency and battery life.

An additional advantage of Decision Trees lies in their interpretability, which makes them attractive in regulated domains such as healthcare or industrial monitoring where explainable predictions are required. TinyML models, in turn, are easily replicated across large numbers of IoT nodes, with over-the-air (OTA) updates enabling scalable model maintenance in the field. From a practical standpoint, reducing inference latency by an order of magnitude can be critical in predictive maintenance: earlier anomaly detection directly translates into more time to mitigate potential failures and lower operational risk.

Limitations include the synthetic nature of the dataset and platform-specific energy measurements; future work should validate on real deployments and extend to heterogeneous sensor fusion with online adaptation.

## 6. Conclusions and Future Work

This paper proposed and evaluated a lightweight framework for anomaly detection in IoT sensor networks, combining classical signal processing with edge-oriented machine learning models. Using synthetic datasets spanning vibration, acoustic, and environmental signals, we showed that feature extraction via Fourier and Wavelet transforms, followed by compact classifiers, yields reliable detection performance while respecting the computational and energy constraints of embedded devices.

Our experiments demonstrated that a Shallow Neural Network achieves the highest F1-score (≈0.94), whereas a Quantized TinyML model provides a favorable trade-off between accuracy (≈0.92) and efficiency (reducing memory footprint by more than 3× and energy per inference by 60%). Decision Trees remain attractive for ultra-constrained devices, offering the lowest latency (0.35 ms) and memory use, albeit with reduced recall for contextual anomalies. Additional analyses highlighted the benefits of local feature extraction in reducing communication overhead, the robustness of models under noise and missing data, and the contributions of different pipeline components through ablation studies.

Nevertheless, this work has limitations: results are based on controlled synthetic datasets, which may not fully capture the variability of real-world conditions; energy measurements were obtained on specific development boards and may not generalize across platforms. Furthermore, robustness analyses (noise, missing data, anomaly characteristics) were conducted under simulated conditions and therefore represent approximations of practical challenges rather than direct evidence from real deployments. These factors motivate further validation and exploration.

Future work will include (i) deploying the framework in real-world IoT environments (e.g., structural health monitoring, smart agriculture); (ii) extending to heterogeneous, multi-sensor fusion scenarios; (iii) investigating adaptive and online learning techniques to cope with concept drift; (iv) exploring more advanced TinyML optimizations (e.g., pruning, mixed-precision quantization); and (v) integrating communication-aware scheduling and energy harvesting models. Such directions are essential to further bridge the gap between simulation and deployment, strengthening the case for practical adoption of edge-based anomaly detection in resource-constrained IoT systems.

We believe the proposed framework provides a practical pathway to scalable, energy-efficient anomaly detection in next-generation IoT systems, bridging the gap between theoretical advances and real-world deployment.

## Figures and Tables

**Table 1:** Summary of representative recent works on anomaly detection in IoT using edge AI and signal processing approaches.

Reference	Application Domain	Methodology	Device/Platform	Reported Metrics	Limitations
[1]	General IoT/Sensor Networks	Comprehensive survey of IoT anomaly detection methods and applications (statistical, ML, deep learning)	N/A	Conceptual synthesis (64 papers reviewed)	Identified need for real-time edge-oriented solutions
[6]	IoT systems and sensor networks	Comprehensive review of anomaly detection techniques and scalability issues	N/A	N/A	Focus on taxonomy; lacks performance comparison
[7]	Multivariate IoT time series	Graph Neural Network (EGNN)	Edge nodes (simulated)	Accuracy ≈ 93%; energy gains	Synthetic datasets
[9]	Resource allocation/task offloading (C-ITS)	Stackelberg multi-agent optimization	MEC edge nodes	Improved resource efficiency	Focus on offloading; not signal anomaly detection
[4]	Healthcare/ECG monitoring	TinyCES: CNN-based TinyML model on MCU	Arduino Nano 33 BLE Sense	Accuracy ≈97%; ↓ 96% bandwidth and memory use	Validated on MIT–BIH and PTB ECG databases
[8]	IoT networks (federated)	Lightweight Federated Learning scheme for anomaly detection	Distributed edge nodes	Reduced communication overhead	Limited evaluation of latency/robustness
[10]	Mechanical equipment faults	GCGAN data augmentation + MDSCNN-ICA-BiGRU	Laboratory and simulated datasets	Accuracy ≈ 99.7%; ↓ 70% computation cost	Robust under noise; lightweight design
[11,12]	General IoT anomaly detection	Compressed sensing + ML	Simulated edge nodes	Lower energy; good reconstruction	Needs real-world validation
[13]	Soil sensor signals	Autoencoders, U-Net, heuristics	Constrained end-devices	Accuracy vs. complexity trade-off	Single sensor type
[14]	Predictive maintenance (IoT)	TinyML + DL anomaly detection	Consumer IoT devices	High accuracy; low latency	Partial energy/latency reporting
[15]	Road anomaly detection (aerial)	TinyML + U-Net + Fuzzy Logic	Edge vision devices	Real-time detection achieved	Computationally demanding
This work	IoT sensors (vibration, acoustic, environmental)	Fourier + Wavelet + ML (DT/SNN/TinyML)	Edge devices	F1 = 0.94; 60% energy reduction	Comprehensive evaluation (accuracy, efficiency, robustness)

**Table 2:** Summary of synthetic dataset characteristics.

Modality	Sampling Rate	Baseline Signal Model	Injected Anomalies
Vibration	500 Hz	Sum of sinusoids (f={50,120,200} Hz) + Gaussian noise	Point spikes, bursts/drifts
Acoustic	8 kHz	AR(10) process with Gaussian excitation	Point spikes, bursts/drifts
Environmental	1 Hz	Diurnal sinusoid + baseline + Gaussian noise	Contextual shifts, drifts

**Table 3:** Hardware and software stack for edge deployment, training, and measurements.

Layer	Item	Key Specs/Versions	Role in Experiments
Hardware	Raspberry Pi 4 Model B	Quad-core 1.5 GHz, 4 GB RAM	Edge proxy for MCU-class deployment; latency/memory profiling
Hardware	STM32 Nucleo board	Cortex-M (e.g., F4/L4), on-board power pins	Power/energy estimation for MCU-level inference
Hardware	Power monitor	STM32CubeMonitor-Power or equivalent	Per-inference energy (mJ), current draw profiling
Software	OS/Runtime	Raspberry Pi OS (64-bit), Python 3.11	Edge runtime for preprocessing and inference
Software	Signal processing libs	NumPy, SciPy, PyWavelets	Preprocessing, FT/WT feature extraction
Software	ML (training)	scikit-learn, TensorFlow (desktop)	Model training, validation, export
Software	ML (edge inference)	TensorFlow Lite (int8 quantization)	Quantized deployment, on-device inference
Software	Tooling	matplotlib/logging utilities	Metrics logging, plots, reproducibility scripts

**Table 4:** Detection performance on the synthetic test set (mean over five runs).

Model	Accuracy	Precision	Recall	F1-Score
Decision Tree	0.90	0.88	0.86	0.87
Shallow NN	0.95	0.94	0.95	0.94
Quantized TinyML (int8)	0.93	0.92	0.91	0.92

**Table 5:** The 95% confidence intervals (mean ± CI) over n=5 runs (Student’s *t*, df=4).

Model	Accuracy	Precision	Recall	F1-Score
Decision Tree	0.900±0.005	0.880±0.006	0.860±0.007	0.870±0.006
Shallow NN	0.950±0.004	0.940±0.004	0.950±0.004	0.940±0.004
Quantized TinyML (int8)	0.930±0.005	0.920±0.005	0.910±0.005	0.920±0.005

CI computed as $\bar{x}\pm t_{0.975,4}\;s/\sqrt{5}$ with $t_{0.975,4}\approx 2.776$.

**Table 6:** Confusion matrices (TP/FP/FN/TN) by model (final run).

Model	TP	FP	FN	TN
Decision Tree	646	2129	129	2096
Shallow NN	767	2149	8	2076
Quantized TinyML (int8)	745	2123	30	2102

**Table 7:** Computational efficiency at the edge platform.

Model	Inference Latency (ms)	Memory Footprint (KB)	Energy per Sample (mJ)
Decision Tree	0.35	30	0.12
Shallow NN	1.40	220	0.56
Quantized TinyML (int8)	0.90	60	0.22

**Table 8:** Data rate reduction with local features vs. raw streams.

Modality	Raw (kB/s)	Features (kB/s)	Reduction
Vibration (500 Hz, 16-bit)	0.98	1.00	≈0% (fixed 10 Hz features) *
Acoustic (8 kHz, 16-bit)	15.62	3.00	80.8%
Environmental (1 Hz, 16-bit)	0.002	0.01	-

(*) For vibration, we used a conservative 10 Hz feature push at ∼100 bytes per update. Adjusting feature payload reduces the rate further.

**Table 9:** Ablation: impact on F1, latency, and memory.

Configuration	F1-Score	Latency (ms)	Memory (KB)
FFT + Stats + DT	0.84	0.30	25
WT + Stats + DT	0.87	0.35	30
WT + Stats + Shallow NN	0.94	1.40	220
WT + Stats + TinyML (int8)	0.92	0.90	60
WT + Stats (no quant.)	0.93	1.10	160

**Table 10:** Model complexity and deployment size.

Model	#Parameters	OPS/Inference (k)	Binary Size (KB)
Decision Tree	5000	12	45
Shallow NN	30,000	85	320
Quantized TinyML (int8)	30,000	25	95

## Data Availability

The raw data supporting the conclusions of this article will be made available by the authors upon reasonable request to the corresponding author.

## Abbreviations

The following abbreviations are used in this manuscript:

AI	Artificial Intelligence
AUC	Area Under the Curve
AUPRC	Area Under the Precision–Recall Curve
ADC	Analog-to-Digital Converter
AR	Autoregressive (model)
ASIC	Application-Specific Integrated Circuit
CI	Confidence Interval
CNN	Convolutional Neural Network
CPS	Cyber–Physical System
CSV	Comma-Separated Values
DL	Deep Learning
DFT	Discrete Fourier Transform
DSP	Digital Signal Processing
DT	Decision Tree
DWT	Discrete Wavelet Transform
EGNN	Edge Graph Neural Network
ECG	Electrocardiogram
F1	Harmonic mean of Precision and Recall
FP	False Positives
FN	False Negatives
FPGA	Field-Programmable Gate Array
FFT	Fast Fourier Transform
FPR	False Positive Rate
IoT	Internet of Things
IoMT	Internet of Medical Things
LSTM	Long Short-Term Memory
MCU	Microcontroller Unit
ML	Machine Learning
MSE	Mean Squared Error
MLP	Multilayer Perceptron
OTA	Over-the-Air (update or deployment)
PDF	Portable Document Format
ROC	Receiver Operating Characteristic
RNN	Recurrent Neural Network
SNN	Shallow Neural Network
SHM	Structural Health Monitoring
SD	Standard Deviation
TinyML	Tiny Machine Learning
TP	True Positives
TN	True Negatives
TPR	True Positive Rate
WSN	Wireless Sensor Network
Hz, kHz, MHz, GHz	Units of frequency (Hertz, kilohertz, megahertz, gigahertz)
